# Diagnosis and Treatment of Patellar Tendon Gouty Tophus: A Case Report

**DOI:** 10.1055/s-0039-1692675

**Published:** 2019-06-18

**Authors:** Theodoros Bouras, Maulik Gandhi, Andrew Barnett

**Affiliations:** 1 Department of Sports Knee Surgery, The Robert Jones and Agnes Hunt Orthopaedic Hospital, Oswestry, United Kingdom

**Keywords:** gout, patellar tendon, tophaceous, deposits, treatment

## Abstract

The main aim of this case report is to thoroughly describe the steps of diagnosis and treatment in the rare incidence of patellar tendon gouty tophus. The case of a 53-year-old man manual worker who was treated with open excision of the lesion, following failure of extended medical treatment with rheumatological input, is presented. Surgical treatment led to full restoration of the patient's knee function. Open or arthroscopic surgery is a viable option for the unusual case of intratendinous patellar gouty deposition if the patient fails medical management. Medical treatment should still be the mainstay.

Gout is the most prevalent form of inflammatory arthritis, affecting primarily middle-aged men. 1 It is characterized by the deposition of monosodium urate (MSU) crystals in and around joints and other tissues. Hyperuricemia, elevation of serum uric acid levels, is the most common cause of gout. Clinical manifestations can present as recurrent acute attacks of severe pain, or chronic inflammation affecting peripheral, small and large joints with tophaceous intra- or extra-articular deposits. 2 Common sites of soft tissue deposits are the Achilles tendon and the flexor tendons of the hand. 3 4 5 Within the knee, the menisci and popliteus tendon followed by cruciate ligaments, quadriceps tendon, and prepatellar bursa are the most usual locations. However, patellar tendon deposits are less frequent and have been rarely reported. 3 6 We present a case of gouty tophus within the patellar tendon which required surgical excision after 2 years of nonoperative management. To our knowledge, this is the only case report in the literature describing failed rheumatological input for gouty tophus of the patellar tendon requiring surgical excision for recalcitrant symptoms.

## Case Report

### Clinical Presentation

A 53-year-old man manual worker with a 13-year history of gout in his right hallux presented to his general practitioner with right knee pain, stiffness, and giving way with no history of trauma. He had been taking allopurinol for 11 years. He drank 10 to 12 units of alcohol per week and his body mass index was 24.4. On examination, he had anterior knee pain and crepitus was felt from the patellofemoral joint. He had a range of motion from 0 to 90 degrees.

### Imaging

**Fig. 1:**
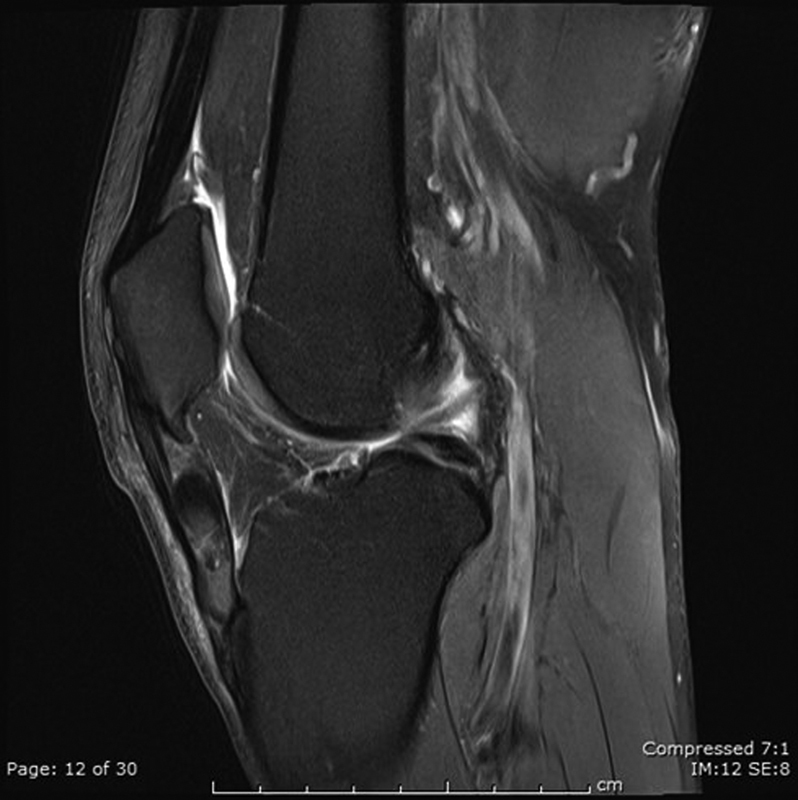
Sagittal magnetic resonance imaging fat suppressed image.

A magnetic resonance imaging (MRI) was performed and reported a grossly abnormal patella tendon showing heterogenous characteristics with areas of architectural distortion and altered signal in all sequences. The appearances were not typical for a tendinosis but more in keeping with findings seen in gout ( Fig. 1 ).

### Orthopaedic Assessment

**Fig. 2:**
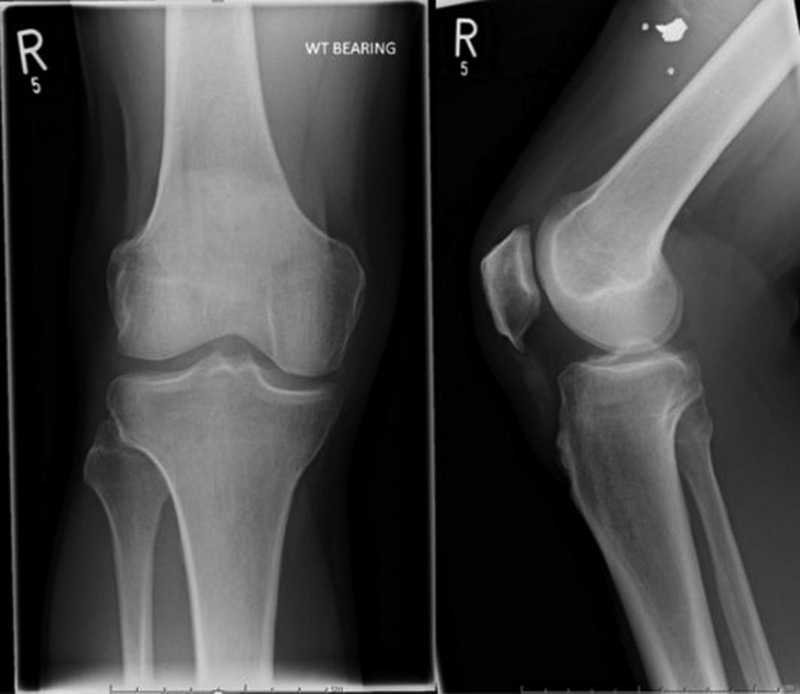
Plain anteroposterior and lateral radiographs.

The patient was commenced on anti-inflammatory medication in addition to his regular allopurinol and referred to an orthopaedic knee surgeon. At assessment, he was found to be significantly compromised by his knee. He was unable to ride a pushbike, walk with his dog, or even get out of a chair. Plain films showed calcification within his patellar tendon ( Fig. 2 ).

### Further Management

**Fig. 3:**
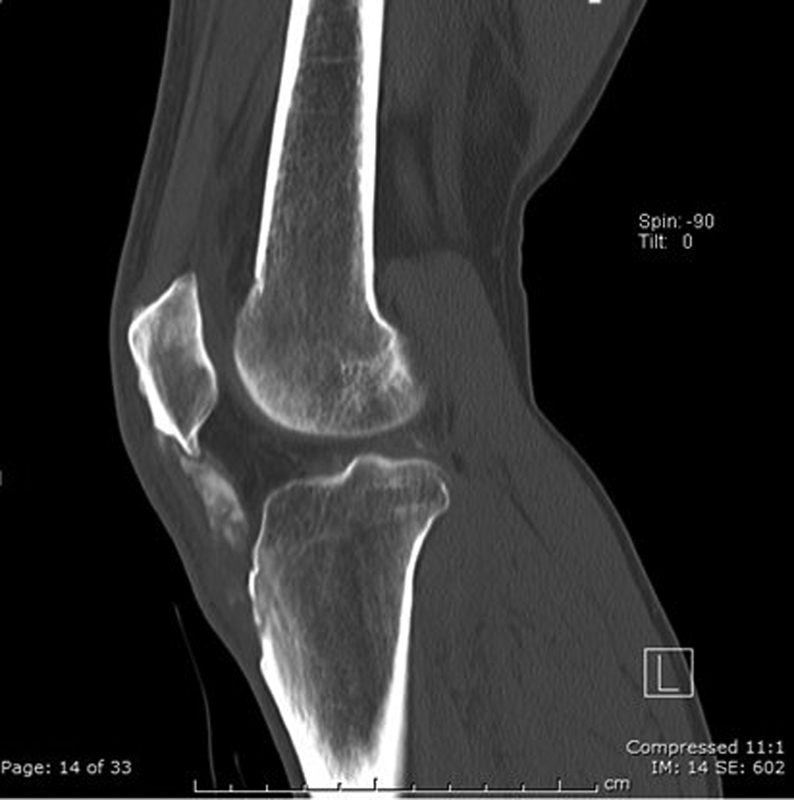
Computed tomography sagittal plane image.

A multidisciplinary discussion with rheumatology and radiology consultants confirmed that the likely diagnosis was a tophaceous gouty deposit within the patella tendon. His uric acid level was 560 μmol/L (above the target of <300 μmol/L set by the British Society for Rheumatology [BSR]) 7 and estimated glomerular filtration rate was 52 mL/min. He was referred to rheumatology who advised increasing the dose of allopurinol. A subsequent ultrasound (US) scan showed the superficial fibers of the patellar tendon relatively intact, but within the deep fibers, there were multiple hyperechoic areas with distortion of the tendon architecture. There was no significant cyst. A computed tomography (CT) scan demonstrated a markedly thickened patellar tendon with areas of mineralization within the tendon itself ( Fig. 3 ). He was seen in a complex knee clinic with three consultant orthopaedic knee surgeons present. With the patient being fully informed about the risk of surgery and patellar tendon weakening and disruption, he was added to the list for open surgical excision of gouty tophus 18 months from initial presentation. His medical management of 200 mg/d of allopurinol had brought his uric acid down to 366 μmol/L with no side effects, but this was still above the BSR guidance. Subsequently, his allopurinol was increased to a daily dose of 300 mg.

### Surgical Management (25 Months from Presentation)

**Fig. 4:**
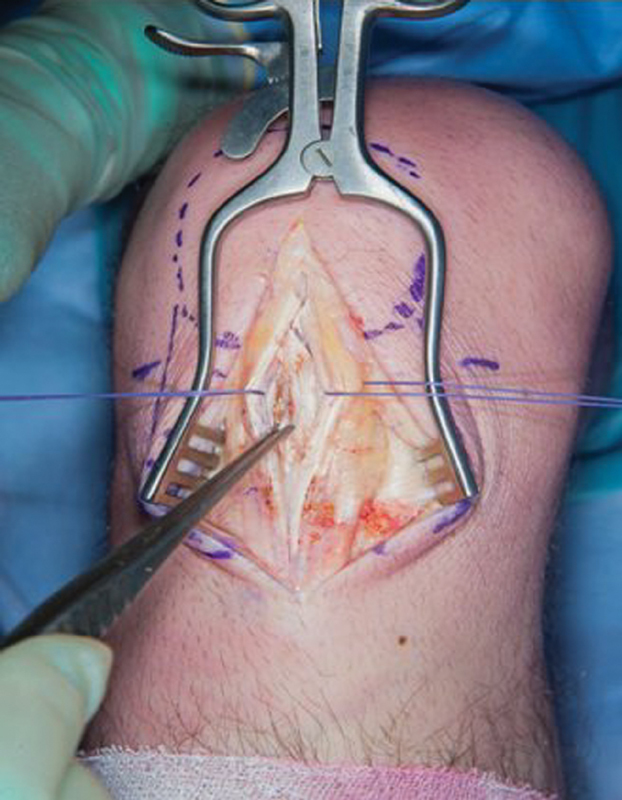
Intraoperative clinic photo showing gouty tophus within the patellar tendon.

**Fig. 5:**
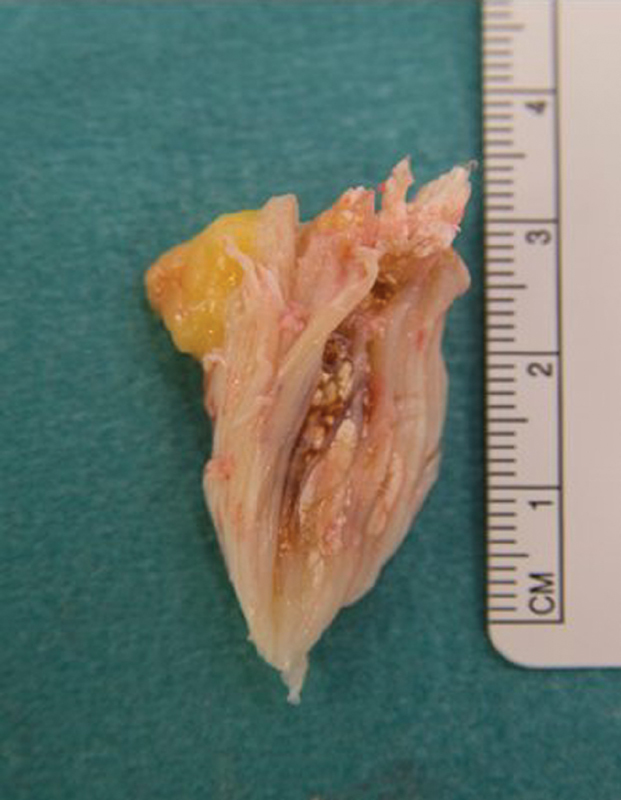
Excised tissue consisting of gouty tophus within patella tendon. *Histology report:* Macroscopic: fibrous tissue measuring 38 × 15 × 10 mm. Slicing reveals chalky white deposits. Microscopic: there are features of a gouty tophus present. There is marginal giant cell accumulation and organization by granulation tissue. Outside this, there is hemosiderin deposition, suggesting that the lesion has been traumatized.

Surgery was performed with the tourniquet inflated at 300 mm Hg for 20 minutes. A midline skin incision was utilized, and the paratenon visualized and incised longitudinally. It was then developed as a definite layer following which the patellar tendon was encountered. The patellar tendon was incised longitudinally and stay sutures placed on either side of the tendon. With gentle traction, the deeper diseased tendon along the lower half of the patellar tendon could be exposed and excised with sharp dissection ( Fig. 4 ). The excised tissue was sent for histology ( Fig. 5 ). Finally, a bony spur was encountered, and this was excised with a nibbler. Postoperatively, the patient was allowed to fully weight bear, at his level of comfort. At 6-week review, the wound had healed with no postoperative complications .The patient was able to perform a straight leg raise and manage a full range of pain-free knee movement. His Oxford Knee score was 45.

## Discussion

The most important finding in this case report is that interstitial gouty tophi of the patellar tendon, despite unusual, can cause debilitating symptoms and can prove difficult to diagnose and treat. Genetic and dietary factors, alcohol consumption, obesity and metabolic syndrome, medications, and renal disease are additional factors to hyperuricemia that increase the risk of gout. 1 2 Two studies reported that symptoms were imitating patellar tendonitis, misdirecting the absolute treatment. 8 9 A recent study reported a case series of gouty involvement of the extensor mechanism of the knee that was mimicking an aggressive neoplasm. 10 In another recent study, the authors had to perform incisional biopsies, following enigmatic imaging findings, to obtain a definitive diagnosis. 11 Imaging modalities include MRI, US, CT, and dual energy CT (DECT), while plain X-rays remain the first line of investigation in the clinical setting. 6 12 13 Joint aspiration still remains the gold standard for the diagnosis of gout with demonstration of MSU crystals or tophus on polarized light microscopy. The 2015 American College of Rheumatology and the European League Against Rheumatism classification criteria for gout allowed diagnosis in luck of concurrent acute symptomatic episode. 4 6 14 The mainstay of treatment is conservative for both intra- and extra-articular gouts. An updated pathway was recently published from the British Society of Rheumatology to the National Institute for Health and Care Excellence guidelines. 7 Most recent studies on current and future therapies for gout have reported new medication combinations toward better and more absolute medical treatments. 15 16 Extra-articular gout presentation has been well described both in upper and lower limbs. 3 6 17 18 However, patellar tendon remains a rare site. To our knowledge, there are seven case reports published so far reporting intrapatellar gouty tophus. Open resection was described in two of them, 8 11 arthroscopic resection and US-guided needle barbotage in one, respectively. 13 14 15 16 17 18 19 One case reported a 42-year-old man triathlete with a family history of gout. He was initially diagnosed with patellar tendonitis. Final diagnosis was established with US-guided aspiration. He was treated conservatively. 9 The other two cases reported the diagnoses of patellar gouty tophi using DECT. 20 21 We contribute to the literature by identifying a case of symptomatic recalcitrant gouty tophi which failed rheumatology-led medical management to manage hyperuricemia. We conclude that surgery can be performed successfully to remove the symptomatic tophus even in the context of hyperuricemia.

## Conclusion

Medical management should be first line in patients who have gout-related symptoms. If the patients fail medical management, then surgical treatment open or arthroscopic can be performed at a later setting without compromising final outcome.
